# Supplementary material for: Amoebocytes facilitate efficient carbon and nitrogen assimilation in the Cassiopea-Symbiodiniaceae symbiosis
Source: Proc Biol Sci. 2020 Dec 16;287(1941):20202393. doi: 10.1098/rspb.2020.2393 (PMC7779505; doi:10.1098/rspb.2020.2393)
Supplement: Supplementary methods and results [file rspb20202393supp1.docx]

Amoebocytes facilitate efficient carbon and nitrogen assimilation in the *Cassiopea*-Symbiodiniaceae symbiosis

Niclas Heidelberg Lyndby, Nils Rädecker, Sandrine Bessette, Louise Helene Søgaard Jensen, Stéphane Escrig, Erik Trampe, Michael Kühl, Anders Meibom

# Supplementary information

Supplementary table 1 | NanoSIMS isotopic enrichment data summary. Treatment IDs correspond to the 5 treatments detailed in the methods section and Figure 1 of the main text. Delta values of ^13^C and ^15^N represent mean values ± SE calculated from all replicates of a given treatment/tissue area as described by equation 1 and 2 in the methods section.

| Treatment ID | Tissue area | Replicates | 𝛅^13^C | 𝛅^15^N |
| --- | --- | --- | --- | --- |
| *i* | *Symbiodinium* | 112 | 26 ± 2 | 3 ± 2 |
| *ii* | *Symbiodinium* | 97 | 1864 ± 93 | -1 ± 1 |
| *iii* | *Symbiodinium* | 81 | 3759 ± 203 | 3 ± 6 |
| *iv* | *Symbiodinium* | 83 | 51 ± 3 | 11069 ± 511 |
| *v* | *Symbiodinium* | 104 | 4675 ± 201 | 10116 ± 573 |
| *i* | Amoebocytes | 26 | 5 ± 2 | 2 ± 2 |
| *ii* | Amoebocytes | 14 | 116 ± 14 | 4 ± 2 |
| *iii* | Amoebocytes | 15 | 424 ± 45 | 2 ± 5 |
| *iv* | Amoebocytes | 18 | 14 ± 2 | 1689 ± 94 |
| *v* | Amoebocytes | 20 | 534 ± 36 | 2397 ± 143 |
| *i* | Epidermis | 20 | 2 ± 3 | 1 ± 1 |
| *ii* | Epidermis | 11 | 167 ± 1 | 2 ± 2 |
| *iii* | Epidermis | 11 | 111 ± 16 | 2 ± 2 |
| *iv* | Epidermis | 16 | 19.1 ± 2 | 794 ± 107 |
| *v* | Epidermis | 18 | 181 ± 8 | 989 ± 45 |

## Incubation light for pulse-labelling experiments

The downwelling spectral irradiance was measured for 3 simultaneous incubations using a calibrated MSC-15 spectroradiometer (GigaHertz-Optik) and was adjusted to a photon irradiance of 350 µmol photons m^-2^ s^-1^ (PAR, 400-700 nm; equal to 64 W m^-2^ s^-1^). Supplementary figure 1 shows the averaged (visible, 400-700 nm) spectrum used for all incubations in this study.


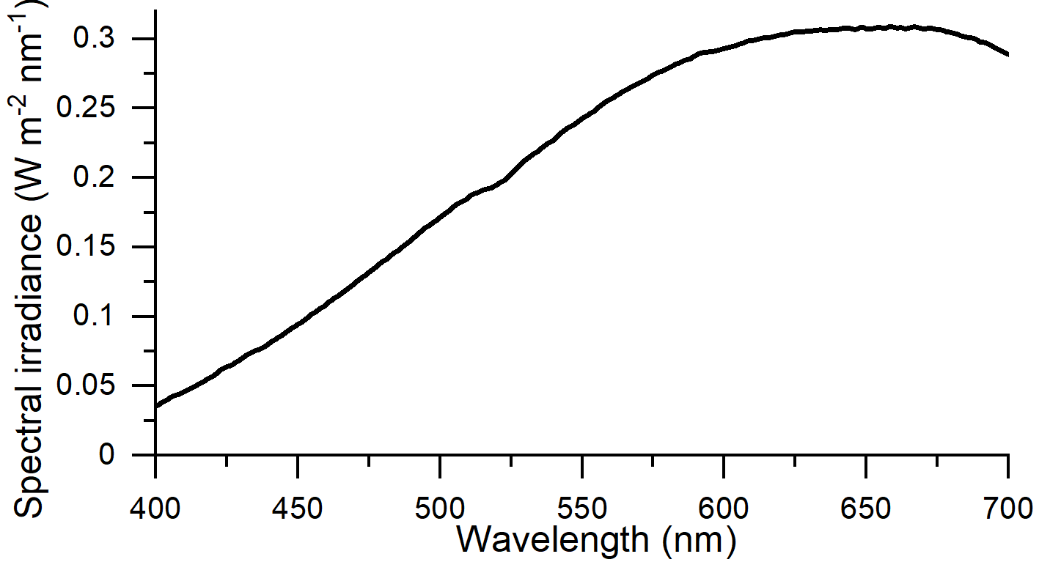


Supplementary figure 1 | Spectrum of lamp used for light incubations in *Cassiopea* pulse-labeling experiments.

## Pulse Amplitude Modulated (PAM) chlorophyll fluorescence imaging

Rapid light curves [RLC; 1] were performed for 17 actinic light intensities at 0-650 µmol photons m^-2^ s^-1^, (2, 20, 33, 47, 69, 96, 128, 155, 178, 208, 242, 290, 339, 396, 469, 554, and 657 µmol photons m^-2^ s^-1^) with an exposure time of 20 seconds per level. The effective quantum yield, $Y(II)$, quantum yield of regulated energy dissipation, $Y(NPQ)$, and quantum yield of nonregulated energy dissipation, $Y(NO)$, were calculated as:

Eq. 1: $Y\left( II \right)=(Fm^{'}-F)/Fm'$

Eq. 2: $Y(NPQ)=1-Y(II)-1/[NPQ+1+qL(Fm/F0-1)]$

Eq. 3: $Y(NO)=1/[NPQ+1+qL(Fm/F0-1)]$

Eq. 4: $Y\left( II \right)+Y(NPQ)+Y\left( NO \right)=1$

where $F$ denotes the fluorescent yield in the presence of actinic light, $Fm’$ denotes the maximum fluorescent yield during a saturation pulse, and $qL$ is the fraction of PSII reaction centers that are open. Data were collected by placing all 3 specimens from a given treatment in the same glass container filled with non-labelled artificial sea water (ASW) at experimental temperature and salinity, and PAM measurements were performed immediately before the isotope pulse label incubations started.

Calculations were performed as regions of interest (ROIs) by selecting the entire umbrella and oral arms (but not the umbrella margin) using the software ImagingWin (v2.41a, Heinz Walz).

## PAM chlorophyll fluorescence imaging

RLC measurements performed for *Cassiopea* sp. samples subjected to white light pulse labelling incubation showed an increasing relative electron transfer rate (rETR) up to around 300-400 µmol photons m^-2^ s^-1^ (Supplementary figure 2). The rETR started to decline at light intensities above 400 µmol photons m^-2^ s^-1^, indicating photoinhibition above this irradiance level. Consequently, a saturating photon irradiance (PAR, 400-700 nm) of 350 µmol photons m^-2^ s^-1^ was used for all incubation experiments (see methods).


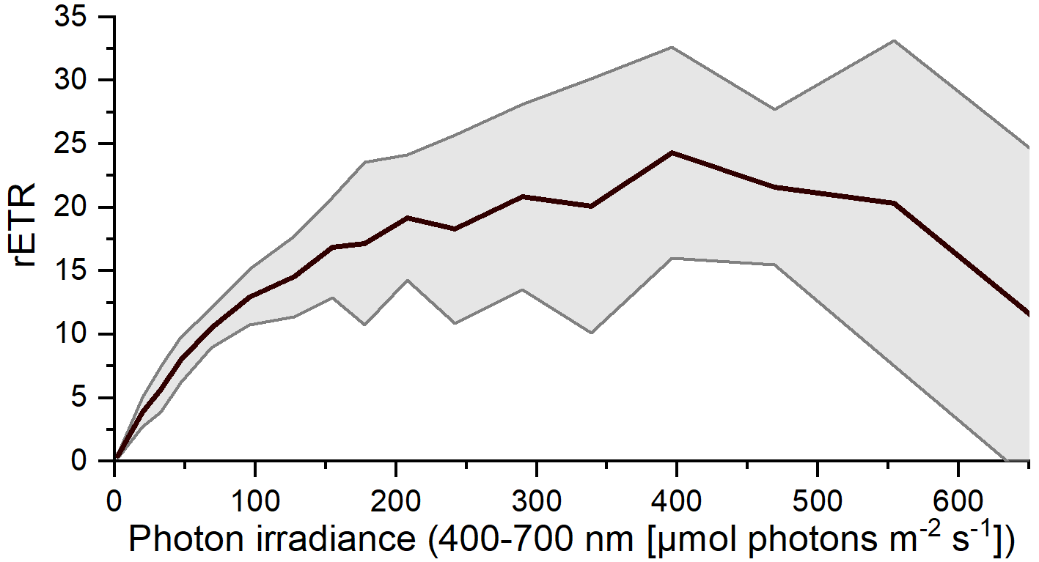


Supplementary figure 2 | Mean relative electron transport rate (rETR) for white light incubated treatments prior to pulse labelling. Thick black line indicates mean rETR for jellyfish pooled across 6-h white light incubation treatments (*N* = 5), and grey shade representing upper and lower 95% confidence limits, respectively.


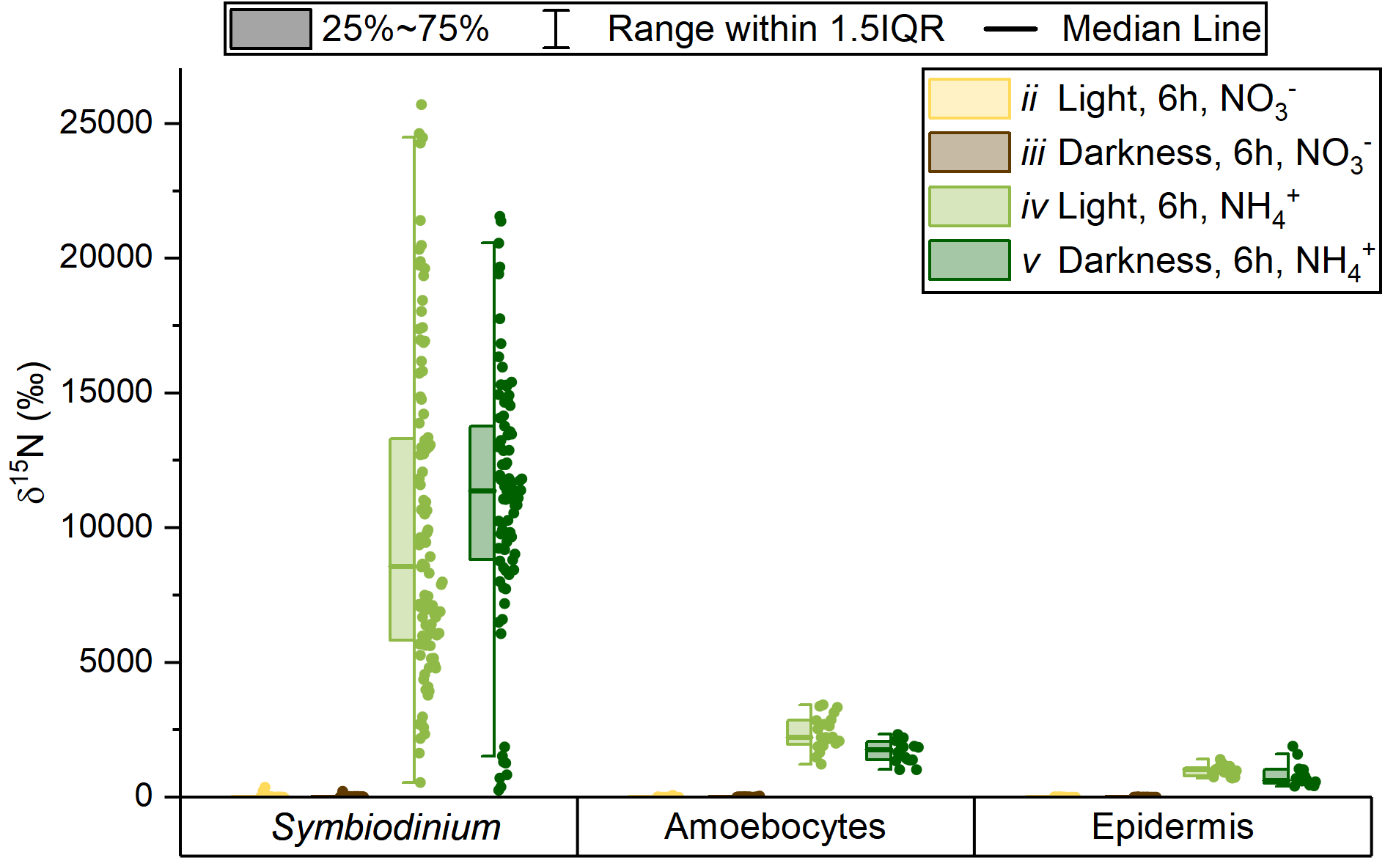


Supplementary figure 3 | Boxplot and data distribution of observed ^15^N enrichment levels in dinoflagellate symbionts and *Cassiopea* sp. host tissue for specimens incubated with ^15^N-nitrate (treatment *ii* and *iii*) and ^15^N-ammonium (treatment *iv* and *v*) 6 h in light (treatment *ii* and *iv*) and 6 h in darkness (treatment *iii* and *v*). The box in plots show the middle 50% interquartile range (IQR) of data with indicated median, and whiskers the range within 1.5 interquartile range (Q1-1.5*IQR or Q3+1.5*IQR).

## Molecular analyses of endosymbiotic dinoflagellates

Living specimens of *Cassiopea* from the aquarium in Denmark were brought back to EPFL to establish which genus of Symbiodiniaceae they hosted.

### DNA extraction

Four *Cassiopea* specimens in different sizes (5-20 mm) were used for DNA extraction. The specimens had prior to extraction been kept frozen at -20°C and were slowly thawed in an open container with ice before further processing. The DNeasy PowerSoil Pro Kit (ref # 47014, Qiagen) was used to extract DNA from the individual samples, and the corresponding handbook for the DNeasy PowerSoil Pro Kit was followed to prepare the samples, with the exception of step 2, in which a Precellys 24 homogenizer (Bertin Instruments) was used with 5500 RPU for 2×20 s at room temperature.

The DNA concentration of the extracts was analysed by applying 1 µl of elution from each sample in a NanoDrop 1000 spectrophotometer (Witec AG). The remaining extracts were stored at -20°C until further analyses.

### PCR amplification

Three genera of dinoflagellate endosymbionts of the family of Symbiodiniaceae [formerly clades; 2] were targeted using nuclear 28S rDNA-specific primers for *Symbiodinium* sp. (formerly clade A; SymA 28S-1F/SymA 28S-1R), *Cladocopium* sp. (formerly clade C; SymC 28S-1F/SymC 28S-1R), and *Durusdinium* sp. (formerly clade D; SymD 28S-2F/SymD 28S-1R). All primers were designed by Yamashita H, Suzuki G, Hayashibara TKoike K [3] except for the forward primer for *Durusdinium* sp., which was modified for increased sensitivity to the following sequence SymD 28S-2F: 5’ AAA GCG CAA GCT TCT TGT C 3’.

Grown cultures of *Symbiodinium* sp. (CCMP2467), *Cladocopium* sp. (CCMP2466), and *Durusdinium* sp. (CCMP2556) were used as positive controls. Strains were purchased from the National Center for Marine Algae and Microbiota (Bigelow Laboratory, East Boothbay, Maine, USA) and cultured in F/2 medium in artificial seawater (35 ppt) at 24°C and 12/12 day/night cycle.

Polymerase chain reactions (PCR) were performed in independent triplicates and the PCR reaction mix consisted of 1x MyTaq Reaction Buffer, 0.4 µM of each forward and reverse primer, 0.1U µl^-1^ MyTaq^TM^ DNA Polymerase (Bioline) and 25 ng of template DNA in 50 µl final volume.

The PCR conditions included an initial 94°C denaturation step for 5 min, followed by 30 cycles of 94°C for 30 s, 54°C for 30 s and 72°C for 25 s, and then a final 72°C elongation step for 5 mins. Absence of contaminations was checked by negative controls. All PCR reactions were carried out using a Biometra TRIO thermocycler (Analytik Jena, LabGene). PCR products were visualized by agarose gel electrophoresis.

## Symbiont identity

The use of three different nuclear 28S rDNA-specific primers for *Symbiodinium* sp., *Cladocopium* sp., and *Durusdinium* sp., respectively, showed that the *Cassiopea* sp. used in this study only hosted the genus *Symbiodinium* sp. Positive controls in form of Symbiodiniaceae cultures of the genera *Symbiodinium* sp., *Cladocopium* sp., and *Durusdinium* sp. was also positive for their respective sets of primers.

1 Ralph, P. J., Gademann, R. 2005 Rapid light curves: A powerful tool to assess photosynthetic activity. *Aquat Bot*. **82**, 222-237. (doi:10.1016/j.aquabot.2005.02.006)

2 LaJeunesse, T. C., Parkinson, J. E., Gabrielson, P. W., Jeong, H. J., Reimer, J. D., Voolstra, C. R., Santos, S. R. 2018 Systematic Revision of Symbiodiniaceae Highlights the Antiquity and Diversity of Coral Endosymbionts. *Current Biology*. **28**, 2570-2580 e2576. (doi:10.1016/j.cub.2018.07.008)

3 Yamashita, H., Suzuki, G., Hayashibara, T., Koike, K. 2011 Do corals select zooxanthellae by alternative discharge? *Mar Biol*. **158**, 87-100. (doi:10.1007/s00227-010-1544-z)
